# The Role of Pan-Immune-Inflammation Value in Determining the Severity of Coronary Artery Disease in NSTEMI Patients

**DOI:** 10.3390/jcm13051295

**Published:** 2024-02-25

**Authors:** Zeki Cetinkaya, Saban Kelesoglu, Aydin Tuncay, Yucel Yilmaz, Yucel Karaca, Mehdi Karasu, Ozlem Secen, Ahmet Cinar, Murat Harman, Seyda Sahin, Yusuf Akin, Ozkan Yavcin

**Affiliations:** 1 Department of Cardiology, Ministry of Health, Elazıg Fethi Sekin City Hospital, Elazıg 23280, Turkey; zeki2387@gmail.com (Z.C.); yucel__karaca@hotmail.com (Y.K.); mehdikarasu@yahoo.com (M.K.); ozllemsecen@hotmail.com (O.S.); seydshn.58@gmail.com (S.S.); yusufkne@gmail.com (Y.A.); ozkanycn@hotmail.com (O.Y.); 2 Department of Cardiology, Erciyes University Faculty of Medicine, Kayseri 38039, Turkey; 3 Department of Cardiovascular Surgery, Erciyes University Faculty of Medicine, Kayseri 38039, Turkey; 4 Department of Cardiology, University of Health Sciences, Kayseri Education and Research Hospital, Kayseri 38100, Turkey; dryyilmaz@hotmail.com (Y.Y.); ahmettcinarr03@gmail.com (A.C.); 5 Department of Cardiology, Fırat University Faculty of Medicine, Elazıg 23119, Turkey; mharman20@hotmail.com

**Keywords:** non-ST elevation myocardial infarction, pan-immune-inflammation value, coronary artery disease severity

## Abstract

Background: Even though medication and interventional therapy have improved the death rate for non-ST elevation myocardial infarction (NSTEMI) patients, these patients still have a substantial residual risk of cardiovascular events. Early identification of high-risk individuals is critical for improving prognosis, especially in this patient group. The focus of recent research has switched to finding new related indicators that can help distinguish high-risk patients. For this purpose, we examined the relationship between the pan-immune-inflammation value (PIV) and the severity of coronary artery disease (CAD) defined by the SYNTAX score (SxS) in NSTEMI patients. Methods: Based on the SxS, CAD patients were split into three groups. To evaluate the risk variables of CAD, multivariate logistic analysis was employed. Results: The PIV (odds ratio: 1.003; 95% CI: 1.001–1.005; *p* = 0.005) was found to be an independent predictor of a high SxS in the multivariate logistic regression analysis. Additionally, there was a positive association between the PIV and SxS (r: 0.68; *p* < 0.001). The PIV predicted the severe coronary lesion in the receiver-operating characteristic curve analysis with a sensitivity of 91% and specificity of 81.1%, using an appropriate cutoff value of 568.2. Conclusions: In patients with non-STEMI, the PIV, a cheap and easily measured laboratory variable, was substantially correlated with a high SxS and the severity of CAD.

## 1. Introduction

Cardiovascular diseases (CVD) are responsible for almost one-third of all deaths worldwide, half of which are caused by ischemic heart disease, continue to be the primary cause of death in developed nations, and are on the rise in developing nations [1]. Although the death rate from CVD decreased by 10.3% between 2007 and 2017, the total number of deaths increased by 21.1% due to a growing population and an aging population [2,3,4].

The underlying pathophysiology of acute coronary syndrome (ACS) is an imbalance between myocardial oxygen supply and demand. The ACS subgroups reflect varied degrees of this mismatch. ST segment elevated myocardial infarction (STEMI) occurs when there is complete vascular blockage, resulting in myocardial injury and necrosis, whereas unstable angina (UA)/non-ST segment elevated myocardial infarction (NSTEMI) is caused by subtotal occlusion [5,6]. ACS occurs as a result of vulnerable (unstable) plaque rupture or endothelial ulceration and is a disease process mainly associated with platelets [7]. Atherosclerotic risk factors, such as inflammation, along with the heterogeneity of plaque histology and the physical pressures exerted on the plaque all have a role in the instability of the plaque [8]. A vulnerable plaque is a plaque with a small fibrous cap and more than 40% necrotic lipid core, and this plaque composition has a high wall tension susceptible to rupture [9]. The clinical manifestation of ACS is determined by several factors, including the thrombotic environment, plaque volume and composition, degree of luminal narrowing, quality of the fibrous cap, and extent of fibrous cap rupture [10]. In addition, platelet aggregation, turbulent blood flow, and coronary spasm are components of the pathophysiology of ACS [11].

Non-ST segment elevated myocardial infarction requires complex pathophysiological mechanisms of systemic/local inflammatory, immunological, and thrombotic systems, which are influential in the formation and progression of atherosclerosis. Among these factors, the study of inflammation and inflammation-based markers has become a prominent topic in recent years [12].

Despite the advancements in interventional treatments and pharmacotherapy, reducing mortality in patients with NSTEMI, the residual risk of cardiovascular events in these patients remains high. Particularly in this patient group, early identification of high-risk individuals is crucial for improving prognosis. Recent focus has shifted towards identifying new associated markers that can better differentiate high-risk patients. With the increasing number of studies showing the importance of inflammation, inflammation markers, and various inflammation determinants (red cell distribution (RDW), neutrophil/lymphocyte ratio (NLR), platelet/lymphocyte ratio (PLR), lymphocyte/monocyte ratio (LMR), systemic immune-inflammation index (SII), etc.) in coronary artery disease (CAD), the relationship between inflammation and CAD is becoming more apparent, and the number of studies is increasing. However, studies with different inflammation markers are needed to find the most accurate of these non-invasive techniques. In this context, studies have evaluated the relationship between markers, such as NLR, PLR, and SII, and CAD severity in NSTEMI patients [13,14,15].

Recently, a new inflammatory parameter, the pan-immune-inflammation value (PIV), has been introduced. Initially proven effective in predicting prognosis in various cancer types, there is increasing evidence of its efficacy in the CVD domain [16,17,18,19]. However, there are no data yet demonstrating the relationship between PIV and CAD severity in patients with NSTEMI. In this study, we hypothesized that PIV is associated with a more severe state of atherosclerotic CAD defined by the SYNTAX score (SxS) in patients with NSTEMI.

## 2. Patients and Methods

A total of 426 patients who applied to our hospital’s emergency department complaining of chest pain and were admitted to the coronary intensive care unit and diagnosed with NSTEMI according to current ESC guidelines were included in this study. This retrospective, cross-sectional study was conducted at a single center.

Patients who had undergone coronary artery bypass surgery (CABG) or who had a history of ACS or percutaneous coronary intervention (PCI) in the last three months, and those found to have normal coronary arteries in coronary angiography were excluded. Furthermore, patients with hematological diseases, malignancies, severe renal disease (estimated glomerular filtration rate [eGFR] < 30 mL/min/1.73 m^2^), liver disease, ongoing infection or chronic inflammatory disease, autoimmune disorders, white blood cell (WBC) count < 4000 cells/µL, and high body temperature (>38°) were also excluded.

Hypertension (HT) was defined as a diagnosis of HT known to be recurrent with blood pressure measurements exceeding 140/90 mm Hg on repeated measurements (at least two) or drug therapy as defined in the guidelines. Diabetes mellitus (DM) was diagnosed in those with a fasting blood glucose of 126 mg/dL, blood glucose > 200 mg/dL at any time, or a history of DM, including those treated with diet, oral medications, and insulin. Hypercholesterolemia has been described in patients with a total cholesterol level > 200 mg/dL and/or low-density lipoprotein cholesterol level > 130 mg/dL or previously diagnosed and treated hypercholesterolemia. Smokers have been defined as regular smokers in the last 6 months.

All participants provided written informed consent before inclusion in the study. The research protocol for this study was approved by the local research ethics committee and is in accordance with the Declaration of Helsinki.

## 3. Laboratory Analysis

Blood samples were taken from all patients between 8 a.m. and 10 a.m. after a 12 h fast prior to coronary angiography for laboratory analysis. Tripotassium EDTA-based anticoagulated tubes were used to collect antecubital venous blood samples. Venous blood samples were taken to measure basic blood variables (such as comprehensive metabolic panel and complete blood count levels). The autoanalyzer (Roche Diagnostic Modular Systems, Tokyo, Japan) was used for all routine biochemical testing. Within 30 min of sampling, hematological samples were stored at 4 °C and analyzed with a Sysmex K-1000 autoanalyzer (SYSMEX K-1000 system, Sysmex, Kobe, Japan). NLR was calculated by dividing the number of neutrophils by the number of lymphocytes. SII was determined by multiplying the platelet count by NLR. PIV was computed by multiplying the SII value and the monocyte count.

## 4. Transthoracic Echocardiography

At the coronary intensive care unit, transthoracic echocardiography was performed on each participant in both the hospitalized and control groups. All measurements were conducted using a machine equipped with a 3.5 MHz transducer (Vivid 5, GE Medical System, Horten, Norway). Two-dimensional echocardiographic measurements were performed to evaluate the left ventricular ejection fraction (LVEF) and valvular pathologies. The Simpson method and color Doppler echocardiography were used to assess the LVEF and valvular pathologies, respectively, in the apical four-chamber view.

## 5. Evaluation of the Coronary Angiography and SYNTAX Score Calculation

All patients received coronary angiography within 24 h of hospitalization. Experienced interventional cardiologists, unaware of the patients’ clinical characteristics and laboratory results, performed the coronary angiography procedure and evaluated the angiography results. Coronary angiography was performed through the radial or femoral artery approach, and the coronary arteries and major branches were evaluated. Routine Judkins catheters were used to cannulate the left and right coronary ostia. The left anterior descending and left circumflex coronary arteries were evaluated in the left caudal, left cranial, right caudal, and right cranial views. Additional views could be taken on the request of the operator. The right coronary artery was evaluated by the left anterior oblique and left cranial views. After coronary angiography, PCI was performed when necessary. When PCI was required, the procedure was performed in the same session, using the same approach route, and treated with standard PCI techniques using 7 French guiding catheters (Launcher; Medtronic). Stenting was performed in the culprit vessel of all patients included in the study. If severe stenosis was present in more than one vessel, the culprit lesion was treated in the first session. The choice of stent type (bare metal or drug-eluting stent) was left to the operators’ decision. Nonionic and low osmolar contrast medium (iohexol, Omnipaque 350 mg/mL; GE Healthcare, Marlborough, MA, USA) was used in all procedures. Immediately after PCI, each patient received 0.9% isotonic solution at a rate of 1 mL/kg/h for up to 12 h.

All patients were treated with oral aspirin (300 mg) and P2Y12 antagonist (ticagrelor 180 mg, prasugrel 60 mg or clopidogrel 300–600 mg) according to the PCI guidelines. Intravenous unfractionated heparin was routinely used as the anticoagulant therapy during the PCI procedures in all patients. Tirofiban was used during PCI according to the operator’s preference.

The anatomical severity of coronary narrowing was quantitatively assessed using the SxS. The coronary tree was divided into 16 segments according to the AHA classification. Coronary vessels with vessel diameters > 1.5 mm were evaluated, and the points assigned to each lesion causing >50% diameter narrowing were summed to calculate the SxS. Each segment was assigned 1 or 2 points according to the presence of disease [20]. This software, the latest online version of which is available on their website, was used to combine the number of lesions with specific weighting factors (ranging from 3.5 for the proximal left anterior descending artery (LAD) to 5.0 for left main branches and 0.5 for smaller branches) based on the amount of myocardium distal to the lesion and the morphological characteristics of each lesion, and the total SxS was calculated [21]. Intra- and inter-observer variability for determining the SxS was 1% and 2%, respectively.

## 6. Statistical Analysis

Data analysis was performed using TurcosaAnalytics v1.0.0 software (Melikgazi, Kayseri, Türkiye) and SPSS (Version 24, SPSS Inc., Chicago, IL, USA). The normal distribution of the data was assessed using the Shapiro–Wilk test and histogram Q-Q plots. Demographic characteristics and laboratory data were categorized according to the SxS and compared using one-way ANOVA for parametric tests with a homogeneous distribution and the Kruskal–Wallis test for nonparametric tests. The Tukey procedure was used for post hoc comparisons on parameters. The Pearson χ^2^ test was used for categorical variables. For assessing the correlation between inflammatory markers and the SxS, the Pearson correlation coefficient for normally distributed data and the Spearman correlation analysis for non-normally distributed data were utilized. ROC curve analysis was used to determine the sensitivity and specificity of the PIV, SII, and NLR values in the high SxS group. The effects of different variables in the high SxS group were calculated with univariate analysis. For the multivariate regression analysis, the parameters with a *p* < 0.05 in the univariate analysis were included in the model. Regression analysis was performed independently for each variable using inflammatory parameters to avoid multicollinearity.

## 7. Results

A total of 426 patients were included in this study; a total of 255 patients were in the low SxS (LSxS) group, 101 patients were in the moderate SxS (MSxS) group, and 70 patients were in the high SxS (HSxS) group. The distribution of the patients’ clinical, demographic, and laboratory parameters across the groups is shown in Table 1.

The patients with a MSxS had a slightly lower LVEF compared to the other groups (*p* = 0.011). Systolic (SBP) and diastolic blood pressure (DBP) were significantly higher in the LSxS group, while heart rate (HR) was higher in the HSxS group (*p* < 0.001). A clinical evaluation showed that the patients in the HSxS group had a higher prevalence of advanced Killip classes (Killip class 3–4) (*p* < 0.001). In the HSxS group, blood creatinine levels were higher, and GFR values were lower (respectively, *p* < 0.001, *p* = 0.006). Troponin and C-reactive protein (CRP) levels were highest in the HSxS group (respectively, *p* = 0.014, *p* < 0.001). Neutrophil levels were highest in the MSxS group, while platelet and monocyte levels were highest in the HSxS group (respectively, *p* = 0.06, *p* < 0.001, *p* < 0.001). Although lymphocyte levels were lowest in the HSxS group, this was not statistically significant (*p* = 0.67). NLR, SII, and PIV were lowest in the LSxS group and highest in the HSxS group (both *p* < 0.001) (Table 1). No significant differences were observed between the groups for the other variables.

The independent determinants of HSxS (SxS > 32) were identified using univariate and multivariate logistic regression analyses. In the univariate analysis, variables such as troponin, SBP, DBP, HR, aspartate transaminase, NLR, SII, and PIV were associated with the severity of CAD. The multivariate logistic regression analysis revealed that DBP, SBP, SII, NLR, and PIV were independent predictors of CAD severity (Table 2). Inflammatory markers (NLR, SII, PIV) were correlated with the SxS (Table 3).

**Figure 1:**
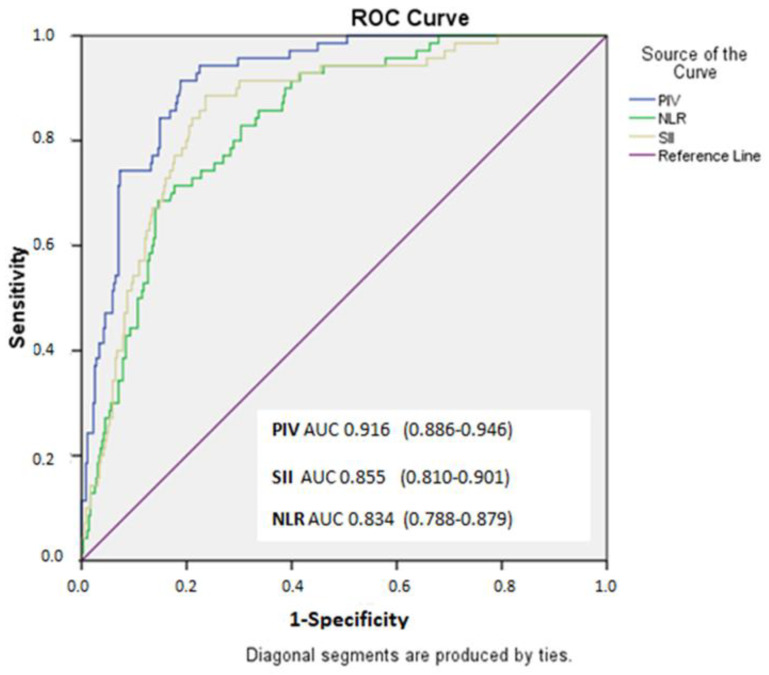
Receiver operating characteristic (ROC) curve for pan-immune-inflammation value as a predictor of high SYNTAX score.

When the ROC analysis was conducted for the SxS patients, it showed that, with a cutoff value of >568.2 for PIV, it predicted the SxS with 91% sensitivity and 81.1% specificity (area under the ROC curve = 0.916 [95% CI: 0.886–0.946], *p* < 0.001). For SII, a cutoff value of 740.8 had predicted the SxS with a sensitivity of 88.5% and a specificity of 76.4% (area under the curve (AUC) = 0.855 (95% CI: 0.810–0.901), *p* < 0.001). For NLR, a cutoff value of 4.01 had 68.5% sensitivity and 85.3% specificity in predicting the SxS in patients with NSTEMI (area under ROC curve = 0.834 (95% CI: 0.788–0.879), *p* < 0.001) (Figure 1).

**Figure 2:**
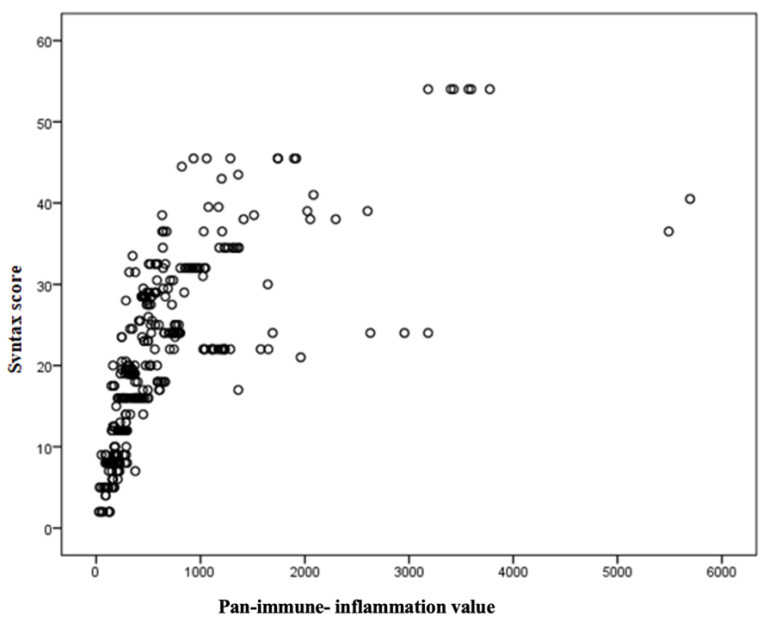
Pan-immune-inflammation value and SYNTAX score have a positive linear association, as observed in the scatter plot.

In NSTEMI patients, the scatter plot in Figure 2 illustrates the relationship between the PIV and SxS. A robust correlation was observed between the PIV and SxS (Spearman’s Rho 0.68, *p* < 0.001) (Table 3 and Figure 2).

## 8. Discussion

This study is the first to demonstrate the association of the PIV with the presence and severity of CAD. Our findings suggest that higher levels of the PIV are indicative of more severe CAD.

There is extensive literature supporting the role of inflammation in CAD. Inflammatory cells, inflammatory proteins, and inflammatory responses from vascular cells play an important role in the pathogenesis of various stages of atherosclerosis, including atheroma initiation and progression, plaque instability and rupture, post-angioplasty, and restenosis [22,23,24]. Endothelial damage occurs due to predisposing factors, such as hemodynamic forces, oxidative stress, and modified lipoproteins [25]. Due to endothelial damage and secreted adhesion molecules, leukocytes begin to adhere to the endothelium [26]. Monocytes, lymphocytes (the balance of TH1/TH2 cells and the cytokines they secrete in the area of inflammation is the driving force of immune-mediated vascular damage), and neutrophils migrate to the subendothelial tissue, and monocytes begin to change into macrophages [27,28]. With the absorption of LDL by macrophages, fatty streaks appear, which is the beginning of the atherosclerotic process [29,30,31]. After this stage, there is more inflammatory cell migration and more LDL accumulation. Together with these cytokines, smooth muscle cell proliferation and collagen tissue increases occur. Collagen synthesis and degradation is a dynamic process [32,33].

The vulnerability of atherosclerotic plaque is thought to be related to underlying pathologies independent of the degree of stenosis of the plaque and forms the basis of the physiopathology of acute coronary event development [33]. With decreased collagen production, fibrous tissue weakens and becomes ready to rupture with enzymes released from macrophages [22]. Plaque rupture causes contact between pro-coagulant lipid content and blood. This causes further inflammation, platelet activation, blood clotting, and vasomotor reactions. If there is too much luminal occlusion to prevent blood flow, ACS occurs [22,34].

The SYNTAX score is a tool used to assess the complexity of disease based on the anatomy of CAD and the location of lesions in the coronary arteries. This score is utilized particularly in guiding treatment decisions between CABG surgery and PCI. Studies have shown that the SxS is a good indicator of adverse cardiovascular events, including cardiac death, myocardial infarction, and revascularization of the target lesion [35].

Aktürk et al. [36] demonstrated that, in patients with NSTEMI undergoing PCI, the SxS was an independent predictor of in-hospital major adverse cardiac events (MACE), stent thrombosis, in-hospital mortality, and non-fatal myocardial infarction. Zuin et al. [37] found a significant relationship between the NLR and the patients’ SxS and its association with 1-year cardiovascular mortality in STEMI or NSTEMI patients treated with PCI within 24 h. Li et al. [14] claimed that new SII biomarkers, including NLR and SII, had better predictive value for MACE and similar predictive value for overall mortality compared to the SxS in NSTEMI patients. Gur et al. [38] demonstrated that a high SII was an indicator of a longer hospital stay and a greater atherosclerotic burden in high-risk patients with ACS in addition to well-known risk factors.

Studies have established atherosclerosis as a chronic inflammatory disease. Inflammatory markers have been frequently used to determine the severity, morbidity, and mortality of CVD. High values of NLR and SII have been reported as determinants of subclinical inflammation and shown to be independent predictors of cardiovascular events and mortality in NSTEMI and STEMI [14,38,39]. Additionally, both markers have been found to correlate significantly with the SxS, suggesting their predictive ability for severe atherosclerosis and mortality in ACS patients [38,40]. Our study also identified a relationship between the NLR, SII, and SxS measured in NSTEMI patients, reaffirming the prognostic importance of these markers in assessing the severity of CAD in this patient group.

The pan-immune inflammatory ratio, though a relatively recent addition to the literature, has limited studies regarding its use in patients with CAD. Şen et al. [39] showed that impaired coronary flow after PCI was associated with high pre-procedural PIV values. Bayramoğlu et al. [40] demonstrated the association of the PIV with no-reflow in STEMI patients post-PCI. Cetinkaya et al. [41] reported an independent association of the PIV with the development of CIN in NSTEMI patients. Liu et al. [19] found that post-PCI PIV values in STEMI patients were associated with long-term MACE. The literature on the relationship between the PIV and SxS in NSTEMI patients is scarce. Our study found that those with higher PIV values had a higher SxS, suggesting that patients with more widespread CAD have higher PIV values.

In the initiation, progression, and complication phases of atherosclerotic plaques, four main blood cell components actively participate. Lymphocytes are crucial in every stage of the atherosclerotic process and in regulating the inflammatory response. Lymphopenia may occur due to a reduction in cell production in response to physiological stress, redistribution at the tissue level, or cell apoptosis [42]. Neutrophils, the most common subtype in the blood, play a significant role in managing inflammatory responses. In advanced stages of atherosclerosis, neutrophils exacerbate tissue damage and inflammation through their proteolytic enzymes, superoxide radicals, mediators, cytokines, growth factors, and direct invasion of smooth muscle and endothelial cells, altering plaque stabilization. Additionally, it has been found that, in atherosclerotic lesions, the neutrophil count is inversely proportional to the number of smooth muscle cells and the thickness of the fibrous cap. Conversely, a positive correlation has been found with the area of the necrotic core, lesion size, and plaque vulnerability [43,44,45,46,47,48].

Arterial thrombosis is largely determined by platelets, which also play a role in inflammation and atherogenesis, contributing to thrombus formation. Activated platelets have been proven to contribute to the development of atherosclerotic lesions and atherothrombosis. Numerous studies have indicated the crucial role of platelets in the development and extent of CAD [49,50].

Previous studies have suggested that a marker like the SII, which combines three different inflammatory parameters (neutrophils, lymphocytes, and platelets) into a single index, could be a more sensitive parameter in predicting the host’s immune and inflammatory status [51,52,53,54,55,56]. The addition of monocytes, considered the core of local inflammation, to these three inflammatory parameters led to the creation of a new inflammatory parameter named the PIV, encompassing approximately all blood cell types [56].

The inflammatory index known as the PIV has recently been studied more. It includes four inflammatory cell types: neutrophils, lymphocytes, monocytes, and platelets. It can be assumed that one-component inflammatory markers of these cells are better predictors than two-component inflammatory markers (e.g., PLR and NLR), and this idea is strengthened by the increasing number of studies in the literature. This speculation can also be made for three-component markers such as SII. Neutrophils are strong influencers of all atherosclerotic plaque processes, both directly by invading the plaque and indirectly by the proteolytic enzymes and arachidonic acid they emit [43,44]. With endothelial damage, fatty streaks (lipid-laden monocytes and macrophages (foam cells) and T lymphocytes) begin to form with the involvement of monocytes and lymphocytes [45]. With the direct involvement of mononuclear cells (monocytes and lymphocytes) and/or cytokines, proteolytic enzymes, and growth factors, the damage shifts in that direction, and the atherosclerotic plaque progresses [45]. Lymphopenia occurs by a mechanism such as decreased cell production due to physiological stress, tissue-level redistribution, or cell apoptosis [42]. With increased lymphocyte apoptosis in the atherosclerotic plaque, plaque development progresses, and its stabilization is impaired. Platelets regulate lymphocyte activation through complex pathways and have an effect on the function of lymphocyte subpopulations [46]. They also contribute to the migration and proliferation of smooth muscle cells and monocytes by releasing their granules, such as thrombin, cytokines, and growth factors [45,47,48]. Previous studies have shown that a marker such as SII may be a sensitive parameter in predicting immune and inflammatory states in the host [52]. By adding monocytes, which are considered to be the nucleus of local inflammation, to this triple inflammatory parameter, a new inflammatory parameter is obtained that includes almost all blood cell types [57]. This suggests that the PIV may be a more sensitive and comprehensive parameter for predicting immune and inflammatory/anti-inflammatory states in the host.

In this clinical context, the addition of monocyte counts to the PIV led us to speculate that the PIV could have a slightly higher predictive value in assessing the extent of CAD.

Previous studies have shown that SII may be a more sensitive parameter in predicting immune and inflammatory states in the host [51,56]. With the addition of monocytes, which are considered to be a nucleus for local inflammation and are considered to affect the inflammatory/anti-inflammatory cascade at every stage, to this triple inflammatory parameter, a new inflammatory parameter including almost all blood cell types is obtained [57]. This suggests that the PIV may be a more sensitive and comprehensive parameter in predicting immune and inflammatory/anti-inflammatory states in the host.

The results of this study indicate that the PIV is a strong inflammatory marker, like the other aforementioned markers, proving its mettle in predicting the extent of CAD. Larger and multicentric studies are required for a better analysis of all potential determinants.

## 9. Limitations

This study has some limitations. First, a relatively small number of patients were included, and it was a single-center, retrospective study. Second, the PIV levels were only calculated at the time of hospital admission. We were unable to include the medications used in the study population in the analyses. The severity of coronary arteries was evaluated solely based on luminal narrowing in visual coronary angiograms, and we did not provide additional information about the quantitative assessment of coronary artery atherosclerosis. Moreover, although high PIV levels are an effective determinant in establishing the severity of CAD, we cannot claim their influence in directing therapeutic approaches.

## Figures and Tables

**Table 1:** Demographic features and laboratory findings of patients in SYNTAX score groups.

Variables	Low SYNTAX Score (<22) *n*:255	Moderate SYNTAX Score (≤22–32) *n*:101	High SYNTAX Score (≥32) *n*:70	*p*
Age n (q1–q3)	66 (57–71)	65 (55–71)	66 (58–73)	0.46
Men, n (%)	173 (67.8)	81 (80.2)	50 (71.4)	0.07
DM, n (%)	131 (51.4)	56 (55.4)	35 (50)	0.73
HT, n (%)	167 (65.5)	69 (68.3)	44 (62.9)	0.75
Smoker n (%)	154 (60.4)	74 (73.3)	47 (67.1)	0.07
Hypercholesterolemia (n, %)	78 (30.6)	25 (24.8)	19 (27.1)	0.52
Left ventricular ejection fraction (%)	55.5 ± 5.4 ^a^	53.6 ± 6.7 ^b^	54.2 ± 5.3 ^b^	0.011
Systolic blood pressure (mm Hg)	120 (110–130) ^a^	100 (100–110) ^b^	95 (90–100) ^c^	<0.001
Diastolic blood pressure (mm Hg)	80 (70–80) ^a^	60 (60–70) ^b^	60 (60–65) ^c^	<0.001
Heart rate (beats/min)	75 (65–90) ^a^	90 (70–103) ^b^	110 (89–116) ^c^	<0.001
Killip Class				
Class I	220 (86.6)	45 (45.5)	8 (11.4)	<0.001
Class II	31 (12.2)	37 (37.4)	21 (30)	
Class III	1 (0.4)	17 (17.2)	36 (51.4)	
Class IV	0	0	5 (7.1)	
Grace score	118 (96–128) ^a^	153 (133–157) ^b^	170 (157–179) ^c^	<0.001
Glucose (mg/dL)	154.5 ± 76.7	173.8 ± 90.2	150.3 ± 60.4	0.07
Creatinine (mg/dL)	0.87 ± 0.2 ^a^	0.95 ± 0.32 ^b^	1.01 ± 0.38 ^b^	<0.001
GFR (mL/min/1.73 m^2^)	86.8 ± 20.4 ^a^	81.6 ± 26.9 ^ab^	76.7 ± 27.1 ^b^	0.006
Total cholesterol (mg/dL)	168.4 ± 48.3	163.9 ± 39.9	170.1 ± 33.9	0.85
AST	23 (18.5–33) ^a^	28 (21–43.2) ^a^	34 (20–72.5) ^b^	<0.001
ALT	19 (13–26.2)	20 (15–27)	20 (14.7–33.2)	0.28
LDL-C (mg/dL)	125.9 ± 35.7	120.2 ± 35.3	127.5 ± 45.5	0.52
HDL-C (mg/dL)	42.7 ± 9.6	41.3 ± 9.4	45.2 ± 10.4	0.26
Troponin	562 (63–1832) ^a^	542.5 (129.7–2243.5) ^ab^	2133 (230.5–9603) ^b^	0.014
CRP	4.87 (2.07–10)	7.6 (4.7–19.5)	8.5 (4.5–21.5)	<0.001
Neutrophil count (10^3^/µL)	4.37 ± 1.7	14.2 ± 16.1	10.8 ± 3.1	0.06
Lymphocyte count (10^3^/µL)	2.06 ± 0.67	2.1 ± 1	1.57 ± 0.8	0.67
Monocyte (10^3^/µL)	0.6 ± 0.19 ^a^	0.75 ± 0.25 ^b^	1.03 ± 1.1 ^c^	<0.001
Platelet count (10^3^/µL)	227.7 ± 67.7 ^a^	245.5 ± 62.2 ^b^	262.1 ± 76 ^b^	<0.001
NLR	1.8 (1.4–2.5) ^a^	3.5 (2.6–5.8) ^b^	4.8 (3.2–7.5) ^c^	<0.001
SII	418 (310.4–543.2) ^a^	896.1 (627.3–1327.6) ^b^	1286.2 (890.3–1757.9) ^c^	<0.001
PIV	249.8 (171.7–349.1) ^a^	582.1 (464.7–800.8) ^b^	1054.4 (670.1–1569.4) ^c^	<0.001

Abbreviations: ALT, alanine aminotransferase; AST, aspartate aminotransferase; CRP: C-reactive protein; DM, diabetes mellitus; HT, hypertension; HDL-C, high-density lipoprotein cholesterol; GFR, glomerular filtration rate; LDL-C, low-density lipoprotein cholesterol; NLR, neutrophil/lymphocyte ratio; SII, systemic immune-inflammation index; PIV, pan-immune-inflammation value. Kruskal–Wallis test and one-way ANOVA test were used when comparing three groups. Groups with statistically significant differences are labeled with different letters (a, b, c).

**Table 2:** Univariate and multivariate logistic regression analysis showing the independent predictors of the presence of SYNTAX score ≥ 32.

	Univariate Analysis			Multivariate Analysis		
	Odds Ratio	95% CI	*p*	Odds Ratio	95% CI	*p*
Left ventricular ejection fraction (%)	0.979	0.939–1.02	0.308	1.01	0.918–1.126	0.754
PIV ^a^	1.003	1.002–1.003	<0.001	1.003	1.001–1.005	0.005
SII ^a^	1.001	1.001–1.002	<0.001	1.001	1–1.001	0.042
NLR ^a^	1.226	1.138–1.321	<0.001	1.181	1.022–1.364	0.024
Systolic blood pressure (mm Hg)	0.901	0.876–0.927	<0.001	0.88	0.783–0.989	0.033
Diastolic blood pressure (mm Hg)	0.858	0.823–0.896	<0.001	1.208	1.027–1.421	0.022
Creatinine (mg/dL)	0.614	0.1–2921.2	0.91	2.28	0.321–16.1	0.41
CRP	1.005	0.99–1.01	0.263	0.989	0.964–1.015	0.416
AST	1.003	1–1.006	0.046	1.002	0.99–1.005	0.268
Troponin	1	1–1.001	0.004	1	1.000–1.001	0.564
Heart rate (beats/min)	1.063	1.046–1.080	<0.001	1.017	0.981–1.055	0.347

^a^: To prevent multicollinearity, analysis was carried out separately with these parameters. Abbreviations: AST, aspartate aminotransferase; CRP: C-reactive protein; NLR, neutrophil/lymphocyte ratio; SII, systemic immune-inflammation index; PIV, pan-immune-inflammation value.

**Table 3:** Bivariate correlations between inflammatory markers and SYNTAX score.

	r	*p*
PIV	0.68	<0.001
SII	0.53	<0.001
NLR	0.49	<0.001

Abbreviations: NLR, neutrophil/lymphocyte ratio; SII, systemic immune-inflammation index; PIV, pan-immune-inflammation value.

## Data Availability

ZC and SK are the guarantors of the work, and as such, had full access to all the data in the study and takes responsibility for the integrity of the data and the accuracy of the data analysis.
